# Considering the Role of Time Budgets on Copy-Error Rates in Material Culture Traditions: An Experimental Assessment

**DOI:** 10.1371/journal.pone.0097157

**Published:** 2014-05-08

**Authors:** Kerstin Schillinger, Alex Mesoudi, Stephen J. Lycett

**Affiliations:** 1 Department of Anthropology, University of Kent, Canterbury, Kent, United Kingdom; 2 Department of Anthropology and Centre for the Coevolution of Biology and Culture, Durham University, Durham, United Kingdom; Universidade do Algarve, Portugal

## Abstract

Ethnographic research highlights that there are constraints placed on the time available to produce cultural artefacts in differing circumstances. Given that copying error, or cultural ‘mutation’, can have important implications for the evolutionary processes involved in material culture change, it is essential to explore empirically how such ‘time constraints’ affect patterns of artefactual variation. Here, we report an experiment that systematically tests whether, and how, varying time constraints affect shape copying error rates. A total of 90 participants copied the shape of a 3D ‘target handaxe form’ using a standardized foam block and a plastic knife. Three distinct ‘time conditions’ were examined, whereupon participants had either 20, 15, or 10 minutes to complete the task. One aim of this study was to determine whether reducing production time produced a proportional increase in copy error rates across all conditions, or whether the concept of a task specific ‘threshold’ might be a more appropriate manner to model the effect of time budgets on copy-error rates. We found that mean levels of shape copying error increased when production time was reduced. However, there were no statistically significant differences between the 20 minute and 15 minute conditions. Significant differences were only obtained between conditions when production time was reduced to 10 minutes. Hence, our results more strongly support the hypothesis that the effects of time constraints on copying error are best modelled according to a ‘threshold’ effect, below which mutation rates increase more markedly. Our results also suggest that ‘time budgets’ available in the past will have generated varying patterns of shape variation, potentially affecting spatial and temporal trends seen in the archaeological record. Hence, ‘time-budgeting’ factors need to be given greater consideration in evolutionary models of material culture change.

## Introduction

Recent work has highlighted the importance of evolutionary approaches to material culture, which have revealed novel insights concerning the historical processes that influence culture change over time and space (e.g. [1], [2], [3], [4], [5], [6], [7], [8], [9], [10], [11], [12]). As with any evolutionary process, the major factors responsible for the pattern of “descent with modification” to which the archaeological records bears witness, are the existence of variation, the potential for at least some of this variation to be heritable (i.e. via social learning), and the fact that not all existing variants are transmitted in equal numbers to subsequent ‘generations’ [13]. Consequently, it has been recognised that it is imperative to understand the specific causal factors that generate variation during the manual manufacturing process, such as the introduction of copying errors, that result in what can be termed ‘cultural mutation’ [7], [14], [15], [16], [17].

Previous work has established that specific factors, such as motor, perceptive and memory constraints, represent important sources of such cultural mutation, yet only rarely have these been investigated using explicit experimental frameworks. One such study by Eerkens [18] tested empirically the effects of memory limitations on the generation of copying error introduced during the manufacture of 2D objects. Eerkens’ [18] study focused specifically on the mechanisms of variation in cultural artefacts that were produced to be ‘standardised’. Artefactual standardisation may be operationally defined as a relative decrease in variation between assemblages that leads to an increase in similarity or enhanced ‘homogeneity’ between artefact products [19]. In Eerkens’ [18] experiment, each participant copied the two-dimensional shape and form of a variety of target items, such as a business card or a US quarter, using scissors on paper. In one condition, participants reproduced these familiar target items solely from memory, without being presented with them in the study. In a second condition, participants were shown a physical specimen of each item and then asked to reproduce their shape and form (the target forms were removed during the manufacturing task). Participants produced less copying error when they viewed a target form just before the copying task than when they relied purely on long-term memory. These results therefore showed that cultural mutation can occur as a result of the imperfection of long-term memory.

In a second example, Kempe et al. [16] addressed the effect of limitations in human perception on the production of copying error in respect to size differences between artefact copies. It has been known for some time that variation by means of small copying errors is introduced in artefact traditions because of the human visual-perceptive limitation to detect small differences between similar-looking artefacts, especially below ∼3 percent difference in size [18]. This inability to perceive size variation below the threshold of three percent is known in psychophysics as the ‘Weber Fraction’ [16], [20]. Kempe et al. [16] examined the long-term consequence of this perceptual error by transmitting images of handaxes along chains of participants, with each participant instructed to copy exactly the size of the previous participant’s image. As predicted, copying errors accumulated exponentially over these multiple cultural transmission events and eventually generated detectable size variation over the long-term, as had been previously indicated by theoretical modelling and simulation [14].

More recently, we [17] have demonstrated that distinct modes of artefact manufacture may generate differing rates of cultural mutation. Specifically, we experimentally tested Deetz’s [21] hypothesis that artefact traditions involving reductive-only manufacturing processes–as, for example, might be seen in stone tool production–generate inherently higher copy-error rates than circumstances where material may not only be removed, but may also be added back on to an artefact during manufacture. Our experiments, which were conducted under conditions where a number of key factors were held constant across multiple participants in two separate copying exercises, demonstrated that a ‘reductive-only’ mode of copying generates statistically higher shape copying error rates compared with the contrasting situation. Indeed, our results support the premise that copy-error rates are process dependent, and that differing manufacturing processes will have differing ‘mutation’ rates, which need to be considered in cultural evolutionary models.

In sum, such studies have emphasized that the experimental study of parameters surrounding the manual manufacture of material culture is paramount to a complete and scientific understanding of the mechanisms that generate cultural mutation (e.g., copying error) and ultimately affect cultural evolution over the longer term. One potential source of copying error that has not received much attention in the empirical research literature, however, is that of limitations, or ‘constraints’, on the manufacturing time available to produce material artefacts (i.e. the time available to complete a manufacturing task). While it can be intuitively assumed that constraints on production time may have an impact on the generation of copying error, the specific effect of time constraints on cultural variation is not currently known. This is despite growing attention concerning the importance of production time in regards to material culture, technological change and even tool variability [22]. Torrence [23] for example, highlights that the production of manually manufactured tools requires specific quantities of time and energy in the course of overall activities and represents an important factor in material culture as a whole. As Torrence ([23]: 12) states, “time available to complete a task … is a key variable in explaining differences in the structure of hunter-gatherer tool-kits as well as in patterns of procurement, manufacture and discard of artefacts”.

The importance of studying time constraints has also been exemplified ethnographically by Binford’s [24], [25] research of Alaskan mobile foragers. He observed the hunting strategies of the Nunamiut hunters of north central Alaska who survive in extreme (cold) environmental conditions. He collected data on how Nunamiut groups organised their time investment in daily activities, including hunting, craft activities and other subsistence-related activities [24]. Nunamiut hunters gain much of their protein from game hunting migrating caribou herds, and it is important for Nunamiut mobile foragers to maximise their hunting efforts because the extreme environment in which they live is otherwise heavily deprived of food resources [25]. Yet, time availability for artefact production is a limited resource during hunting activities because of the additional time invested in anticipating the high mobility of these animals and the unpredictability of their occurrence. The planning of time invested in tool production is not only important for game hunting preparations. There is also a need to avoid a ‘time conflict’ between tool manufacture and the multiple other essential activities, such as eating, sleeping, travelling, gathering raw resource material prior to tool production, and so forth. Binford [24] observed conflicts between the different subsistence activities, for example, if people invested more time in craft activities, less time was spent on eating and socialising.

The Nunamiut provide an apposite anthropological example of how production time of material cultural artefacts is inevitably a resource that will be limited in the context of mobile foragers. Torrence [23] referred to time limitation during hunting activities as ‘time stress’, leading to daily activities in the life of a mobile forager being carefully organised, or in other words, ‘budgeted’. Binford [25] also acknowledged how tool manufacture required careful (i.e. in-advance) planning and preparation in order to be ‘geared up’ for these difficult game hunting conditions. One further strategy of dealing with such time pressures was to ‘stage’ tool manufacture into different phases, with manufacture taking place at different places and times, and final tool production completed at the hunting stands [24]. Another form of economical scheduling of time resources was the “embedment of tool manufacture and maintenance into other subsistence strategies” ([24]: 12).

Insights provided by Torrence’s [23] and Binford’s [24], [25] research on these ‘time constraints’ affecting tool manufacture have been further incorporated into computational simulation models that investigated the economic factors impacting technological change. The purpose of such models is to consider ‘costly’ technologies over ‘less costly’ alternatives in specific economic terms, such as whether certain technologies can be expected to make greater returns if more time is invested in their manufacture (e.g. [26], [27]). The ecological foraging model by Bettinger et al. [27] can be applied when two different technologies of distinct economical value co-exist as they take up different foraging purposes. Californian Indians, for example, utilized both a cheap and quickly produced ‘self bow’ for leisurely play and rough use, and a more costly but also more effective ‘sinew backed bow’ which required longer production time but was utilized for most difficult game hunting events associated with higher returns ([27]: 544). What these models have in common is that the time spent in tool production is acknowledged to be an important economical factor in tool manufacture.

There are additional ethnographic examples that demonstrate scenarios of how limitations on production time may arise during the manufacture of material culture. Such a circumstance can arise when ecological or economic circumstances require a tool manufacturer to produce a larger quantity of artefacts within the same timeframe, compared to previously smaller quantities of products. For example, research by Layton [28] illustrated that family workshops in the Shandong Province of China, who specialised in wood block printing (among other crafts), endured an economic shift from craft to mass production during the course of the 20^th^ century. Techniques for these crafts were traditionally transmitted within the family from parents to children via patrilineal descent. Initially, woodblock printing was a household-based production model run by family workshops that produced prints for local demand. From the second half of the 20^th^ century, higher quantities of woodblock printing products have been manufactured for commercial purposes. In other words, such family workshops, which previously only supplied domestic and local demand, later faced increased production demand for an expanded clientele of tourists and more widely distributed clients. This constitutes an example of where an increase in production demand initiated an increase in the ‘time constraints’ on production time as greater artefact quantities had to be produced during restricted time availability.

These anthropological examples, and also the economical models by Bettinger et al. [27] and Ugan et al. [26], demonstrate that constraints on manufacturing time are inherent parameters of material culture production. However, despite these anthropological examples demonstrating that time constraints on tool production are present, the question of whether different limitations on tool production time affect the generation of *variation* (i.e. mutation) in artefactual attributes has not been addressed to date. This is despite growing knowledge of the impact that mechanisms of variation, such as copying error, have on evolutionary change in material culture (e.g. [7], [14], [15], [16], [17]).

Here, we investigate experimentally the effects of varying time constraints on copying error during the manual manufacture of cultural artefacts in a laboratory context. One of the advantages of using experiments is the ability to provide specific answers as to whether differing time constraints (such as those seen in the ethnographic examples referred to earlier) can generate differing rates of cultural mutation in artefactual traditions. Moreover, time constraints are specifically tested on copying error related to the metric *shape* of the artefacts. Variation in artefact shape–as opposed to purely size or ‘scale’ variability–is a particularly vital parameter to consider in cultural evolutionary models [29]. Aspects of artefact shape may have specific functional or aesthetic properties [30], [31], [32] and so be subjected to various selective or shape ‘preservation’ biases [33], [34], yet also may be subject to more stochastic drift-like processes, which also create distinct spatial and temporal patterns [4], [35]. Moreover, historically within archaeology, variation in the shape of artefacts has been used as a key variable in temporally and spatially relevant artefact classification schemes [4], [36]. As previously mentioned, recent experimental and computational studies established that the accumulation of copying error can lead to detectable changes in size (i.e. ‘scaling’) parameters in artefacts during the course of long-term cultural transmission [14], [16], [18], but to date, shape mutation has received far less attention [17].

Here, we explore how time limitations affect rates of shape copying errors by manipulating multiple varying ‘time constraints’ on the production time provided. In the experiment, participants copied a target form using a plastic knife and a standardized foam block. A total of 90 participants were divided into one of three ‘time constraints’ (i.e., varying limitations on the production time available): 20 minutes, 15 minutes or 10 minutes. One of the advantages of this design is that we can determine not only whether, but also how, rates of shape copying error alter when constraints on the production time periods are increased systematically. It might, for example, be reasonably hypothesized *a priori* that shape copying error varies proportionately and linearly with production time. That is, shape copying error rate will be lowest for the 20 minute time limit, moderate for the 15 minute time limit, and highest for the 10 minute limit, with statistically significant differences generated with each decrease in time. Alternatively, copying error may not vary proportionately with production time; instead a task specific ‘threshold’ might be the more appropriate manner to conceive of how time budgets affect mutation rates in manufacturing traditions. By testing a variety of different production time periods, the specific impact of time constraints on cultural mutation can be investigated and understood more precisely in respect to whether, and when, rates of cultural mutations change with respect to time constraints.

## Methods and Materials

### Participants

A total of 90 participants were recruited at the University of Kent through a university advertising scheme. All participants in this study were tested in the same laboratory facility. The participant cohort consisted of 45 females (mean age = 23, SD* = *4.14, age range = 18–44 years) and 45 males (mean age = 23, SD* = *3.69, age range = 18–34 years). A reimbursement of £4 for was offered for their participation in the experiment.

### Materials

**Figure 1:**
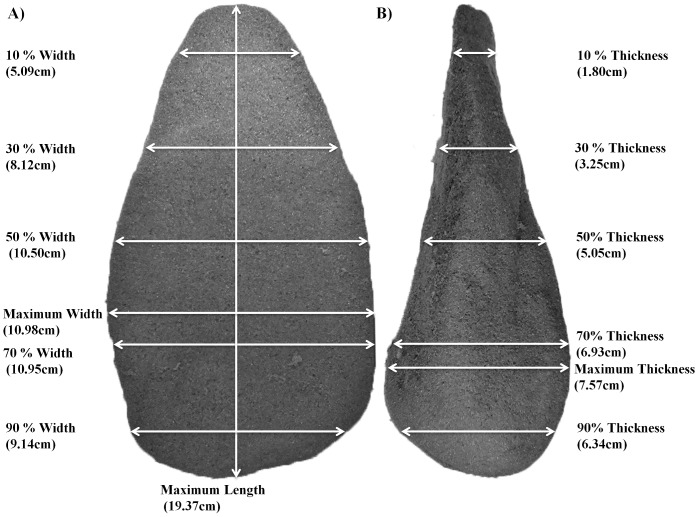
Target form used during experiment.

The ‘target form’ chosen for this experiment was a foam model of an ‘Acheulean handaxe’ (Figure 1). Stone ‘handaxes’ possessing similar form to the model used in our experiment first appear in the archaeological (Palaeolithic) record of Africa from around 1.75–1.5 million years ago [37], [38], but they subsequently appear in Western Europe and large parts of Asia and remain a persistent feature of the archaeological record for over one million years [39], [40], [41]. In the case of hominin stone artefacts and cultural evolution, it is widely contended that the production of these artifacts represents a shift from the manufacture of relatively simple cutting tools (flakes) produced by bouts of knapping not necessarily directed toward the production of deliberate core forms [42] to a situation where tool production was strategically oriented toward *shaping* the residual block of stone [31], [43], [44].

The decision to copy ‘handaxe’ form in foam blocks was made for a variety of reasons. Firstly, the application of real stone knapping was deemed unsuitable for reasons of safety and feasibility, especially given the need to recruit numbers of participants large enough to facilitate sample sizes amenable to statistical analysis. Moreover, the manufacture of handaxes from stone requires levels of skill and experience that are built over months, if not years, of practice [45] and may even result in injury [46]. Conversely, foam ‘handaxes’ are easily manufactured, thus facilitating the immediate recruitment of multiple participants without specialised knowledge. We have previously noted that–somewhat akin to the use of relatively simple ‘model organisms’ in the experimental study of important biological evolutionary processes–the use of handaxe form as a target shape is particularly appropriate in experimental studies of material culture evolution [17]. This is because model organisms, such as fruit flies (*Drosophila spp*.), commonly used in experiments of biological evolutionary processes such as transmission and mutation, tend to possess a variety of characteristics that make them particularly suitable for such experiments, including economy, speed of replication, and controllability (e.g. [47], [48], [49]). The most suitable model organisms, therefore, display some of the complexities of the phenomenon of interest, yet are generally not so complex that they are unwieldy in experimental settings. Similarly, although the production of foam ‘handaxes’ does not necessarily approach the most complex manipulation of form variables that might be required in artefact production, their production certainly requires the manipulation of a multiplicity of integrated aspects of three-dimensional shape, especially in terms of relative length, width and thickness variables (Figure 1). This, of course, does not assume exact equivalency between all aspects of artefact copying in our experiment and the exact details of ancient artefact production, but our experimental set-up does facilitate examination of copying error within the context of varying time constraints while taking advantage of this general comparative “model” framework.

**Figure 2:**
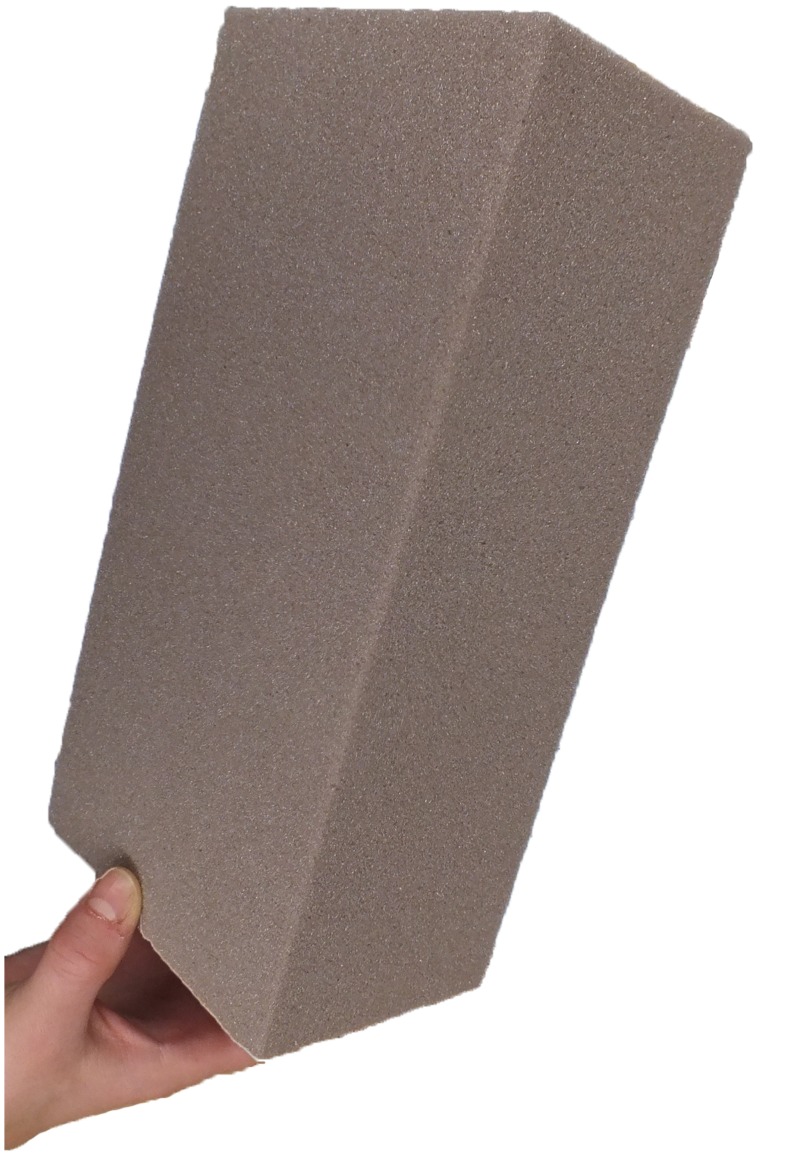
Example of machine-cut foam blocks provided to participants during experiment. Each block measured 22.3×11×7.8 cm.

The foam ‘handaxes’ were produced from standardised blocks of OASIS DRY SEC (dry floral) foam. These machine-cut blocks were obtained from the manufacturer in a standardized format and measured 22.3×11×7.8 cm (Figure 2). The foam consists of a firm porous material which is designed to securely hold the stem of artificial flowers. However, the material is also designed to be malleable so it can be easily modified into desired shapes using simple every day materials such as knives and scissors. The floral foam is, therefore, ideally suited for this experiment, being sufficiently robust to be handled without introducing unwanted shape alterations, but is also easily modified with simple cutting tools. Since the foam manipulation caused a certain amount of foam dust to disperse, participants were also provided with a lab coat to protect clothing, mouth protection and laboratory eye protection glasses.

### Experimental Conditions and Procedure

In this study, the main factor of manipulation was the time constraint under which the participants completed the copying of a target handaxe form. There were three experimental conditions that varied only in the time limit that participants had to produce the handaxe replica: either 20, 15 or 10 minutes. All participants took part only once in the experiment and could not repeat the task in any of the other experimental conditions.

Participants were divided equally and randomly between conditions (n = 30 for each condition). There were equal numbers of 15 females and 15 males in every condition, therefore controlling for any potential visuo-spatial biases resulting from sex differences (e.g. [50], [51], [52], [53], [54]). The majority of participants were right-handed; however, there were left-handed participants in each condition (four individuals in the 10 minute condition and three individuals in the 15 minute and 20 minute condition). Hence, the distribution of left-handed participants (10–13%) and right-handed participants represented that of the general population [42], [55], [56].

Participants in all three conditions were asked to copy the ‘handaxe target’ form (Figure 1). One participant was tested at a time. The participants were instructed to consider the overall shape and form of the model target during the task, but were asked to specifically copy the model handaxe’s *shape*. As an additional incentive to motivate participants, a £20 book voucher was offered to the individual who copied the target form most accurately (produced the replica with the least shape copying error) in addition to the £4 reimbursement.

Depending on which of the three conditions the participants were placed in, the instructions given to participants differed only in the production time provided to complete the copying task (20 minutes, 15 minutes or 10 minutes). Thereafter, each participant was provided with one full minute to examine and handle the target handaxe from different sides prior to beginning their own copy. Once the minute was over, the participants were placed at a table where the experimental task was conducted. All participants were provided with one standardized foam block and a plastic knife (Figure S1) in order to undertake the manufacturing task.

To avoid memory-related confounding effects (see e.g. [18]), participants were permitted to compare the target handaxe with their own replica throughout the experiment. Participants were verbally reminded in five-minute intervals of the time remaining to complete the task. In addition, participants were provided with a digital timer (which counted down the time left to complete the copying task) so they could check the remaining time at any point during the experiment. Participants had only one opportunity to take part and were not able to repeat the experiment in another condition.

Descriptive statistics regarding the time spent in the manufacturing task are summarised in Table 1. Examination of the average times in each condition indicates that the mean times closely approach the maximum time provided in each condition. This shows that, on average, participants utilised the maximum timeframe available in each of the three time conditions to complete the copying task, confirming the validity of our experimental manipulation.

**Table 1:** Descriptive statistics of time spent on completing the manufacturing task.

	Time condition		
	10 min	15 min	20 min
**Mean**	9.96	14.9	19.24
**SD**	0.15	0.33	1.77
**Minimum**	9.4	13.56	13.03
**Maximum**	10	15	20

### Morphometric Procedures and Compilation of the Data Set

**Figure 3:**
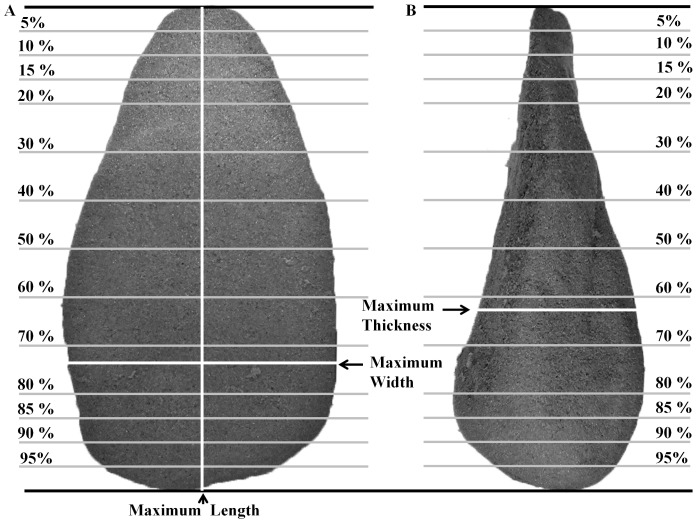
Measurement scheme and the position of measurement gridlines in plan-view (A) and profile-view (B). This grid system provided a total of 42 variables.

For every ‘handaxe’ replica and the ‘target’ model, a set of measurements was obtained for 28 plan-view variables and 14 profile-view variables, creating a morphometric dataset of 42 total variables for each specimen (Figure 3). The measurements were obtained digitally by importing photographic images of each replica into the freely available morphometrics software tpsDig v2.16 [57]. Foam replicas were positioned on a light box to optimally capture the shape outline on the photographs imported to this software. Images were obtained with a Fujifilm DSLR camera that was securely attached to a copystand (30× zoom lens: 24–720 mm). A standardized orientation protocol was applied in order to obtain homologous measurements. The orientation protocol is a slightly modified version of that originally designed by Callow [58] and subsequently applied by Costa [59]. Full details of the orientation protocol can be found in the Text S1. A digital grid was superimposed (Figure 3) onto the images of each foam replica’s plan- and profile-views obtained, which defined the 42 bilateral and lateral measurements (Text S1).

Given that we were specifically interested in shape copying error, data were size-adjusted via use of the geometric mean method [60], [61]. This method of size-adjustment effectively removes size (scaling) variation between specimens by equalizing their volumes, yet retains their relevant shape data [60], [62]. The geometric mean of a series of *n* variables (*a*_1_, *a*_2_, *a*_3_ … *a_n_*) is equivalent to (*a*_1_ × *a*_2_ × *a*_3_ ×… × *a_n_*)*^1/n^*. Simply, the geometric mean is the *n*th root of the product of all *n* variables [63]. The method proceeds on a specimen-by-specimen basis, dividing each variable in turn by the geometric mean of the variables to be size-adjusted. Hence, to implement the method, the geometric mean of each foam replica was calculated separately and thereafter each of the 42 morphometric variables for each specimen were divided by that particular specimen’s geometric mean. The size-adjusted values of the 42 morphometric variables for each of the 90 replicas were subtracted from the equivalent 42 variables of the target model. Thereafter, mean shape error was computed for each of the 42 morphometric variables across the 30 replicas obtained in each experimental condition. It is these 42 mean error rates that were used in the subsequent statistical analyses. The mean shape error rates of the 42 morphometric variables in each of the three time conditions can be viewed in the supplementary Figures S2, S3, S4.

### Statistical Analysis

Data produced in the differing time conditions were first compared statistically using a non-parametric Kruskal-Wallis test, where α = 0.05. This conservative non-parametric test was applied since the resultant shape error data were not normally distributed. For post-hoc comparisons between conditions, we report both the uncorrected Mann-Whitney *U* tests (asymptotic), which some consider valid in the face of a statistically significant Kruskal-Wallis test [64], and the more conservative Bonferroni corrected p’ values, where p’ = pN_pairwise_. All analyses were undertaken in PAST v2.17 [65].

### Ethics Statement

Ethical approval was granted by the University of Kent Ethics Committee. All participants provided written agreement to take part in this experiment by reading and signing a consent form sheet prior the experiment. The data from this study were analysed anonymously. Individual scores and personal information including gender and age cannot be identified from published material since statistical analysis was conducted across the entire sample population.

## Results

**Figure 4:**
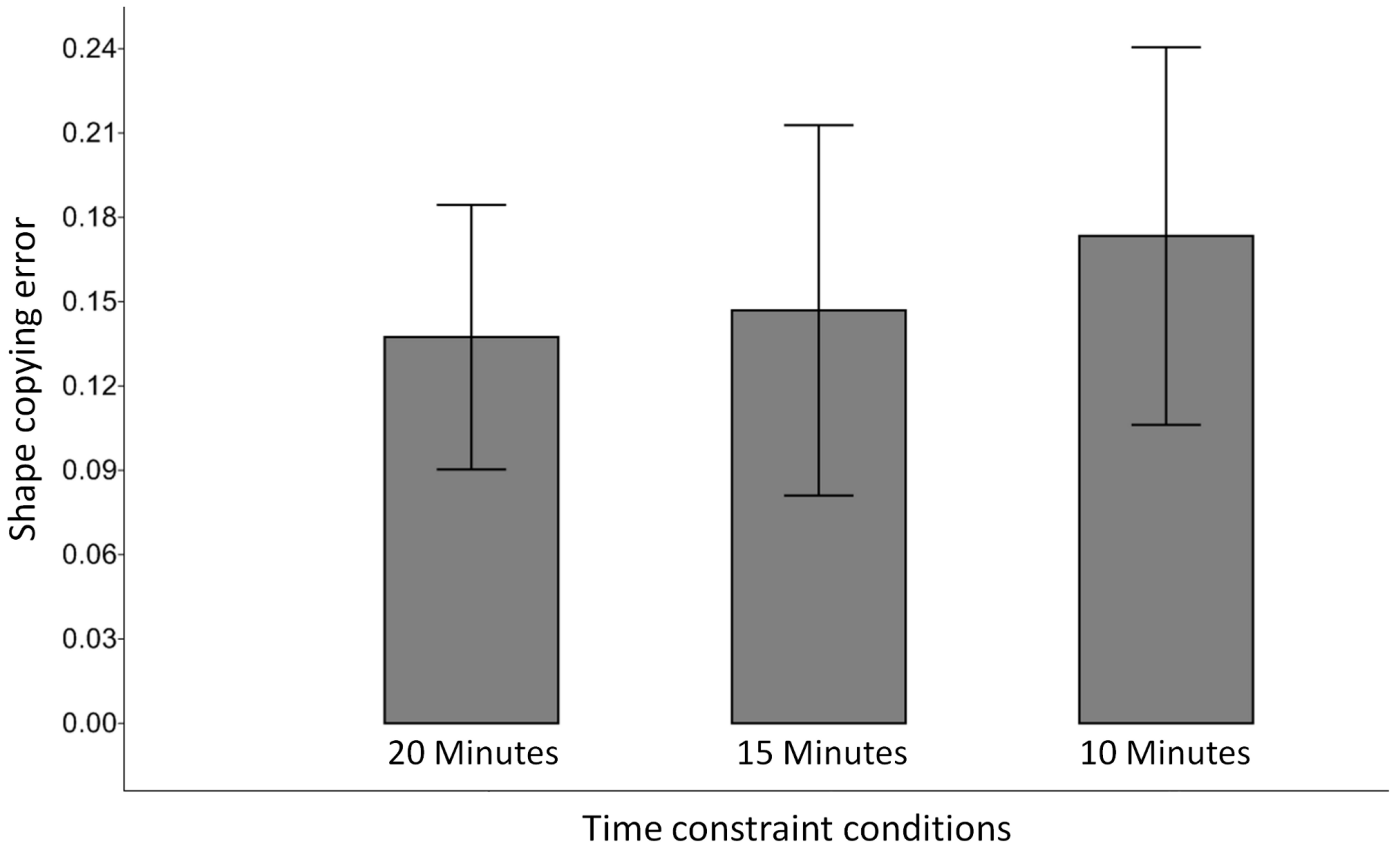
Mean shape copying errors (bars) in the different time constraint conditions. Whiskers show standard deviations (one sigma).

In the 20 minute time condition, participants displayed a mean copying error of 0.137 (SD = 0.047). For the 15 minute time condition an average shape copying error of 0.147 (SD = 0.066) was record. Lastly, an average shape copying error of 0.173 (SD = 0.067) was produced in the 10 minute time condition. These results are illustrated visually in Figure 4.

The Kruskal-Wallis test demonstrated that copy error rates were not significantly equal in all conditions (H = 8.297, p = 0.015). Table 2 shows the results of the post-hoc comparisons. These results indicate no statistically significant differences between the 20 minute condition and the 15 minute condition, either in the raw (uncorrected) comparisons or the Bonferroni corrected comparisons. Similarly, the uncorrected Mann-Whitney *U* test indicated a significant difference between 20 minute condition and the 10 minute condition (*U* = 569, asymptotic p = 0.005), and between the 15 and 10 minute conditions (*U* = 651, asymptotic p = 0.0387). Although this latter result is not statistically significant when Bonferroni correction is applied (p’ = 0.1161), there is still evidence of a statistically significant difference between the 10 minute and the 20 minute condition, even with Bonferroni correction (p’ = 0.0151).

**Table 2:** Mann-Whitney *U* comparisons following Kruskal-Wallis test (H = 8.297, p = 0.015).

	20 min	15 min	10 min
**20 min**	–	0.5867	**0.0050**
**15 min**	1	–	**0.0387**
**10 min**	**0.0151**	0.1161	–

Upper right diagonal = uncorrected (asymptotic) p values, lower left diagonal = Bonferroni corrected p’ values, where p’ = pN_pairwise_.

In sum, in none of our statistical analyses is there evidence for significant differences between the 20 minute condition and the 15 minute condition. Only when time constraints are reduced to 10 minutes (i.e. 50% of maximum) does statistical evidence for differences between conditions emerge. Hence, in statistical terms, shape copying error generated during the course of the experiments changed in a fashion most plausibly explained by the effect of reaching a ‘threshold’.

## Discussion

Ethnographic and computational research on mobile forager societies indicates that the time invested in manual tool production is a vital aspect of hunter-gatherer economy [22], [23], [26], [27]. In fact, anthropological examples of Nunamiut mobile foragers described by Binford [24], [25] illustrate that the presence of a range of subsistence activities as well as unpredictable ecological factors generate ‘constraints’ on the time available for craft activities. Nunamiut foragers have created subsistence strategies to accommodate such constraints, for example, by carefully ‘budgeting’ time [23]. However, constraints on artefact production time can also arise from an alternate anthropological context where manufacturers are faced with the pressure of producing higher quantities of artefacts under limited time availability due to changing economic demands [28].

Our experiment specifically focused on the effect of ‘time constraints’ during manual manufacture on artifactual *shape variation*. This effort to study variation-generating mechanisms is based on recent empirical and computational research studies, which illustrate the importance of studying variation to enhance our understanding of the mechanisms underlying cultural change and evolution [14], [16], [17]. There is growing knowledge that one source of variation, in the form of copying errors, can be introduced during the manual manufacturing process of cultural artefacts, generating between-assemblage variation and potentially leading to visible change over the course of multiple cultural transmission events [14], [15], [16]. Given the import of understanding variation in these terms, time constraints may be an important (yet under-studied) variable that needs to be given greater consideration in cultural evolutionary models. Indeed, since production time is a vital component of manually produced material culture, it is imperative to understand the impact of such time constraints on variation during the manual manufacture of artefacts, especially in terms of potential impacts on cultural mutation rates.

Here, we used an experimental approach to systematically test the effects of gradually increasing time constraints on shape copying error during the production of experimentally produced foam ‘handaxe’ artefacts. In the experiment, all participants were asked to faithfully copy a model ‘handaxe’ target form. In three experimental conditions, the production time was limited either to 20 minutes, 15 minutes, or 10 minutes. Thus, time constraints were increased by shortening the production time systematically by 5 minutes. Overall, the results showed when time constraints are altered by the same amount across conditions, mean levels of shape copying error increased. However, this increase was not sufficient to generate statistically significant differences between the 20 minute and 15 minute experimental conditions. Only when production time was dropped to 10 minutes (i.e. 50% of maximum) did significant differences between conditions emerge. The fact that significance levels in this experiment were primarily driven by a sharp increase in shape copying error in the 10 minute condition indicates that in this condition a ‘critical’ point was reached. In the 10 minute condition high accuracy in the copying was no longer achievable, leading to a sharp increase in copying error rates, at least when compared to accuracy levels obtained when participants had 20 minutes to complete the task.

These results are important given that part of the aim of this study was to determine whether merely reducing production time alone changed copy error rates proportionately across all conditions, or rather, whether the concept of a task specific ‘threshold’ might be the more appropriate manner to model the effect of time budgets on mutation rates in manufacturing traditions. While our results support the hypothesis that decreasing time budgets will steadily lead to increased copy-error rates, in statistical terms, our results more strongly support the idea that shape copying error is best modelled according to a ‘threshold’ effect, below which mutation rates increase more markedly. In our study, this threshold fell somewhere between 15 and 10 minutes, although of course the threshold will vary depending on the task. Future experimental work could help determine whether there is however a linear effect once such thresholds have been reached.

These results generate several implications for the study of spatial and temporal patterns in material culture traditions, perhaps the most obvious of which, is that time budgets will likely be reflected in patterns of variation in archaeological artefacts. Variation in quantitative shape attributes across time and space is a directly measureable feature of the archaeological record [4], [29], [66]. Several factors are likely to influence changing spatio-temporal patterns in such data, including selection factors or cultural biases, as well as stochastic drift (e.g. [2], [15], [33], [35], [67], [68], [69]).

What our results imply, however, is that in addition to these factors, detectable changes in artefactual patterns of spatial-temporal variability may well reflect differing or changing production-time budgets, which themselves, of course, may be subject to processes of selection or cultural drift. Hence, ‘time-budgeting’ factors may need to be given greater consideration in evolutionary models of material culture change.

A further implication arising from these results is the relationship between ‘mutation rate’ in artefactual attributes and patterns of cultural change in evolutionary models. We have previously noted that the appearance rate of new cultural variants may conceptually be linked to potential for evolutionary change ([17]: 137) akin to the concept of ‘evolvability’ in biology ([70]: 587). It must be stressed, of course, that while ‘evolvability’ in these terms might be used to describe the potential for change brought about by selective factors (either natural or cultural), it can also be used to describe potential for the degradation of culturally transmitted traits, leading eventually to their extinction, or cultural ‘collapse’ of a particular tradition. Indeed, although variation is required for selection to operate, and is therefore a prerequisite of cumulative cultural evolution, equally it has been known for some time in biology that ‘mutation load’ is a factor which may ultimately prove fatal to population viability [71]. Hence, in the light of our results, time constraints may be a factor in inducing unsustainable levels of cultural mutation, and so lead to the extinction of particular traditions unless specific cultural (i.e. socially learnable) mechanisms are put in place to check the generation of excessively high mutation rates. In relation to the results reported here, this would imply at least keeping time constraints under certain task-specific ‘thresholds’.

When specifically considering how cultural factors might integrate with these results, one potential insight is that such ‘costs’ may drive a pressure to find cultural means of maximally ‘economising’ production time. One possibility worthy of future consideration in this regard may be the extent to which distinct stages, or components, of manual manufacture (i.e., ‘production stages’) possess their own distinct ‘time budgets’. In other words, where it was described earlier that hunter-gatherer societies compensate for time limitations acting on various subsistence strategies by implementing ‘time budgeting’ strategies [23], [24], [25], the same notion of ‘time budgeting’ may be applicable to the different production stages of the manufacturing process itself. Examples of material culture with conceptually and practically distinct ‘stages’ in production are known widely, for example, in the context of the manufacture of pottery (e.g. [72], [73], [74]: 113–131), basketry [75], [76]; stone tool knapping [31], [77] and textile production [78]. Dynamic ‘time scheduling’ has been described by Torrence ([23]: 12) as “division of time into small parcels which are then juggled according to some set of priorities”. There may, therefore, be a dynamic where such segmented time budgets can be rearranged under varying time constraints in order to strategically optimise production time so that copying error remains low under equivalent time constraints. In the context of artefactual production where the priority is to keep copying error rate low under varying degrees of time constraints, such prospective rearrangement of the ‘time slots’ allocated to manufacture itself may become one possible strategy where different ‘components’ of the manufacturing processes are *distinctively* affected by copying error. In other words, ‘simpler’ as opposed to more ‘difficult’ components of the manufacturing process, may be affected to a lesser degree by copying error under the impact of time constraints. As one possible solution to the optimisation of time stress, such ‘simpler’ production phases could be ‘sped up’ in a fashion whereby shape accuracy can be maintained. Future experimental work may profitably be used to evaluate the effect of differing time budgets on copy-error rates in these terms, and so evaluate these contentions.

Finally, and with the previous point in mind, our results reiterate the importance of using experimental approaches to understand the causes of differing cultural mutation rates in artefactual products [16], [17], [18]. Equally, however, the time provided to participants in order to complete task conditions is a factor that will also need to be taken account of in future experiments of this type.

## Supporting Information

Figure S1 **Dimensions of plastic knives provided to participants.**
(TIF)

Figure S2 **Mean shape-copying error levels in the 20 minute time condition for each of the 42 variables.**
(TIF)

Figure S3 **Mean shape-copying error levels in the 15 minute time condition for each of the 42 variables.**
(TIF)

Figure S4 **Mean shape-copying error levels in the 10 minute time condition for each of the 42 variables.**
(TIF)

Text S1 **Orientation protocol.**
(DOCX)
