# Supplementary figures and images for: Considering the Role of Time Budgets on Copy-Error Rates in Material Culture Traditions: An Experimental Assessment
Source: PLoS One. 2014 May 8;9(5):e97157. doi: 10.1371/journal.pone.0097157 (PMC4014615; doi:10.1371/journal.pone.0097157)

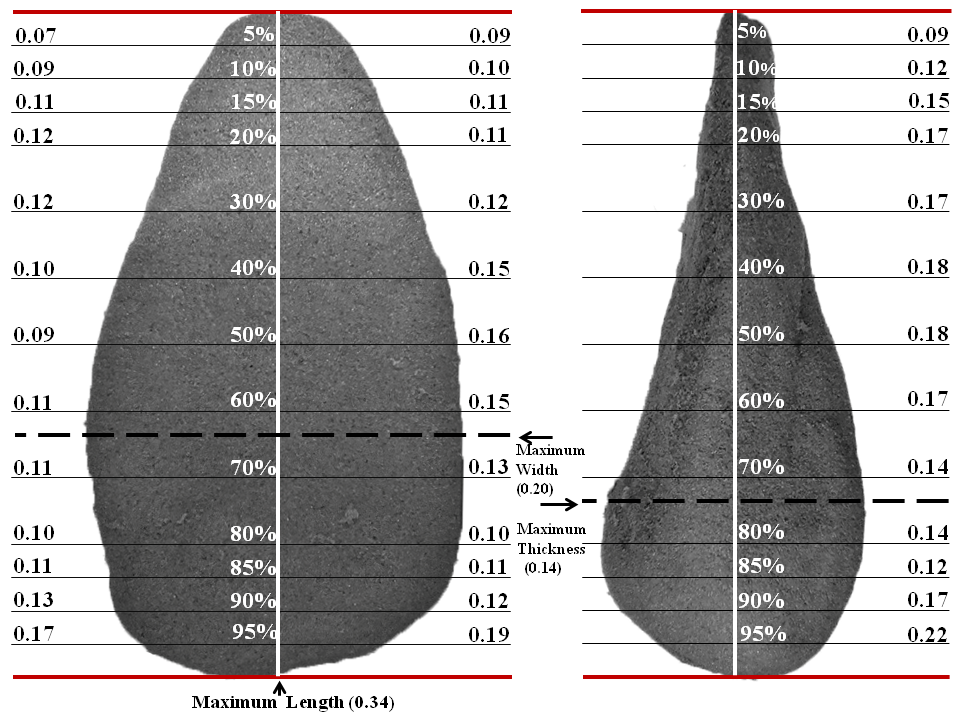

Supplement: Figure S2 — Mean shape-copying error levels in the 20 minute time condition for each of the 42 variables. (TIF) [file pone.0097157.s002.tif]

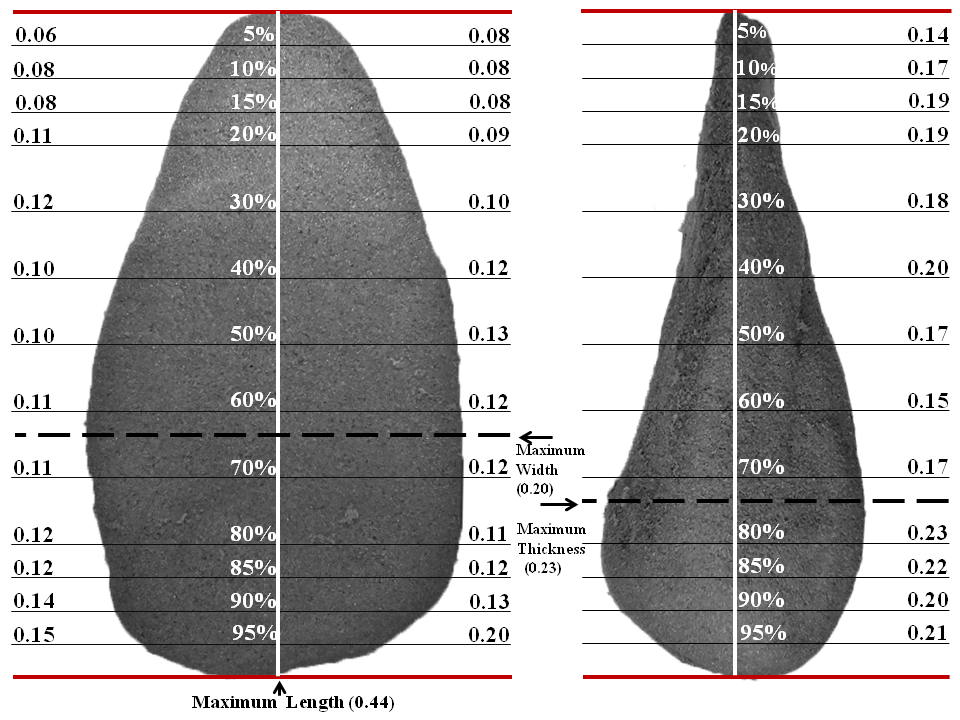

Supplement: Figure S3 — Mean shape-copying error levels in the 15 minute time condition for each of the 42 variables. (TIF) [file pone.0097157.s003.tif]

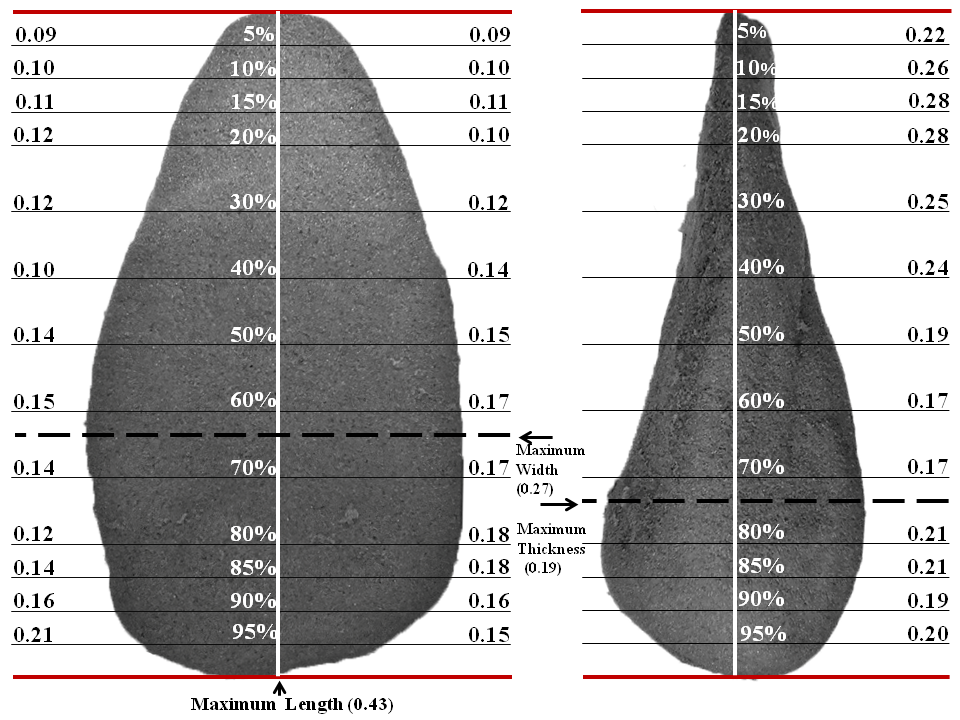

Supplement: Figure S4 — Mean shape-copying error levels in the 10 minute time condition for each of the 42 variables. (TIF) [file pone.0097157.s004.tif]
