# Supplementary material for: Considering the Role of Time Budgets on Copy-Error Rates in Material Culture Traditions: An Experimental Assessment
Source: PLoS One. 2014 May 8;9(5):e97157. doi: 10.1371/journal.pone.0097157 (PMC4014615; doi:10.1371/journal.pone.0097157)
Supplement: Text S1 — Orientation protocol. (DOCX) [file pone.0097157.s005.docx]

**Text S1. Orientation protocol**


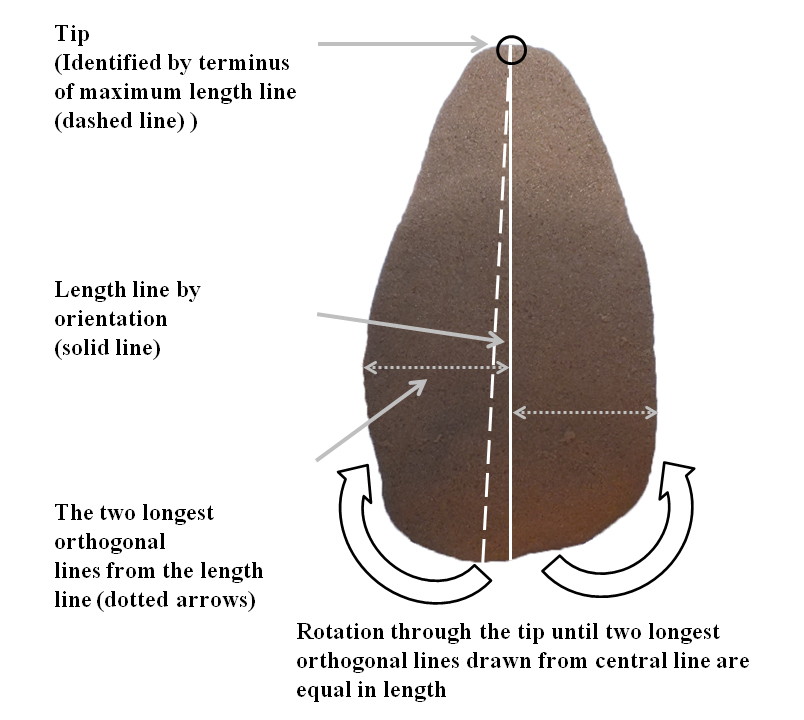


The orientation protocol used in our experiments is a slightly modified version of that originally produced by Callow (1976) and subsequently applied by Costa (2010). The orientation protocol first defines the ‘handaxe tip’ on the plan-view as the terminus of the maximum length line (i.e. maximum dimension) on each ‘foam handaxe’ at the distal end of the handaxe area (indicated by a dashed white line in figure above). Next, each handaxe model is orientated through the ‘tip’ such that the two longest orthogonal lines diverging from a second length line (i.e. ‘length by orientation’) are both equal in length (dotted arrows in figure shown above). The same procedure was repeated for the profile-view. The profile-view for each replica was identified by rotating each handaxe model leftward through an axis of 90 degrees. Hence, all thickness measures in the profile-view are directly orthogonal to the width measurements obtained from the plan-view.

Once each model had been digitally photographed (see main text), lateral and bilateral measurements for the 42 morphometric variables were obtained by positioning a digital grid onto the image of the foam handaxe area so that its central line was placed on top of the length line by orientation. Next, the grid’s upper and lower border lines were fitted to the handaxe replica’s maximum length margins. The grid defined horizontal lines at incremental distances of 10% that diverged bilaterally from the length line by orientation. In order to obtain further shape variation along the ‘tip’ and ‘base’ of the foam handaxe area, additional grid lines were added to the grid at 5 percent, 15 percent, 85 percent and 95 percent of the length line. Once the grid was adjusted to each replica’s shape outline, the image was transferred to tpsDig v2.16 (Rohlf 2010), where the measurements were conducted. For the plan-view, bilateral measurements were recorded from the grid’s central line to the outer boundary of the foam handaxe area. For the profile-view, lateral thickness measurements were obtained by measuring the distance from one handaxe outline to the other. Further measurements were recorded for the maximum length, maximum width (in plan-view) and maximum thickness (in profile-view).

**References**

Callow, P., 1976. The Lower and Middle Palaeolithic of Britain and Adjacent Areas of Europe. PhD thesis, University of Cambridge, Cambridge.

Costa, A.G., 2010. A geometric morphometric assessment of plan shape in bone and stone Acheulean bifaces from the middle Pleistocene site of Castel di Guildo, Latium, Italy. In: Lycett, S.J., Chauhan, P.R. (Eds.), New Perspectives on Old Stones: Analytical Approaches to Palaeolithic Technologies. Springer, New York, pp. 23-41.

Rohlf, F.R., 2010. TpsDig2 v2.16. Stony Brook, N.Y., Department of Ecology and Evolution, State University of New York. Available at: <http://life.bio.sunysb.edu/morph/>
